# 2818. Investigating Urinalysis Criteria that Predict UTI: Impact of age, sex, and urine culture thresholds

**DOI:** 10.1093/ofid/ofad500.2429

**Published:** 2023-11-27

**Authors:** Sonali Advani, Rebecca M North, Nicholas A Turner, Sahra Ahmadi, Julia Denniss, Adero Francis, Anum Hasan, Rachel M Johnson, Faryal Mirza, Sarah Pardue, Meghana V Rao, Yasmin Rosshandler, Helen Tang, Kenneth E Schmader, Deverick J Anderson

**Affiliations:** Duke University School of Medicine, Durham, North Carolina; Duke Aging Center, Durham, North Carolina; Duke University Medical Center, Durham, North Carolina; Wellstar Kennestone Medical Center, Marietta, Georgia; Duke University School of Medicine, Durham, North Carolina; Medical University of South Carolina, Charleston, South Carolina; SOVAH Health-Danville, Sterling, Virginia; Wellstar Kennestone Hospital, Marietta, Georgia; SOVAH health, Danville, Virginia; Sovah Health Danvile, Greensboro, North Carolina; Duke University Hospital, Durham, North Carolina; Wellstar Kennestone Regional Medical Center, Marietta, Georgia; Hospital of the University of Pennsylvania, Philadelphia, Pennsylvania; Duke and Durham VA Medical Centers, Durham, North Carolina; Duke Center for Antimicrobial Stewardship and Infection Prevention, Durham, North Carolina

## Abstract

**Background:**

Urinalysis (UA) results are used by clinicians and laboratories to aid in the diagnosis of UTI. However, optimal UA parameters have not been systematically investigated or validated for different populations. Our objectives were to assess the performance of UA in predicting UTI and to stratify UA performance by age, sex, and culture thresholds.

**Methods:**

We conducted a retrospective cohort study of adult non-catheterized inpatient and ED encounters with paired UA and urine cultures (24 hours apart) from 5 community and academic hospitals in three states (NC, VA, GA) between 01/01/2017 and 12/31/2019. Trained abstractors collected clinical and demographic data for a random sample of patients. Patients were classified as having UTI, asymptomatic bacteriuria or ‘not UTI’ based on IDSA guidelines using signs/symptoms and microbiology data. We evaluated the performance of relevant UA parameters in predicting UTI by assessing sensitivity, specificity, negative predictive value (NPV), and positive predictive value (PPV). We also combined 18 different UA criteria (Figure 1) and used area under receiver operating curves (AUROC) to identify the 5 best-performing models, and stratified results by age, sex, and lower urine culture bacterial threshold (1000 colony forming units).

**Figure:**
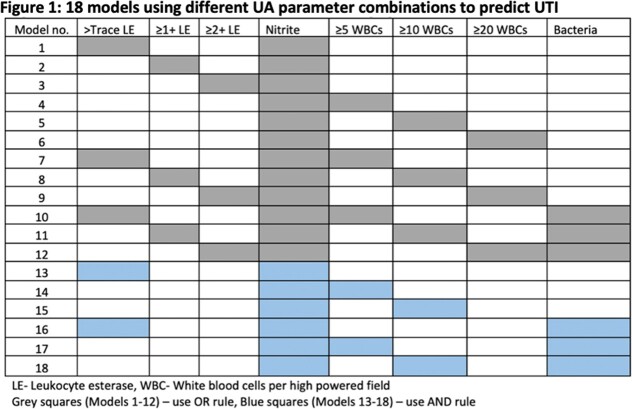

**Results:**

Of 219,338 encounters, 3392 charts were included and reviewed; 723 (21.3%) patients met criteria for UTI. Females and older adults had a higher incidence of UTIs and ASB (P< 0.05; Table 1). Absence of pyuria (or leukocyte esterase) had a high NPV for UTI (Table 2). Combined UA parameters performed better than pyuria alone with regards to NPV and AUROC, specifically models 1 and 5 (Table 3). UA parameters used in these models performed differently based on age, sex and urine culture thresholds, with limited utility in older women and UTIs with lower bacterial threshold (Table 4).

**Figure:**
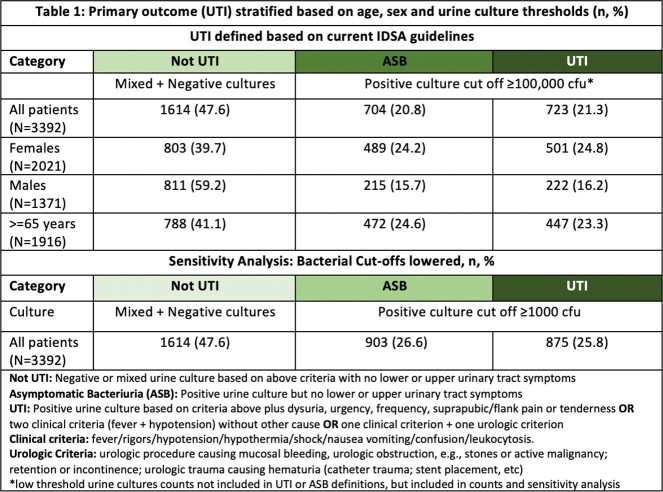

**Figure:**
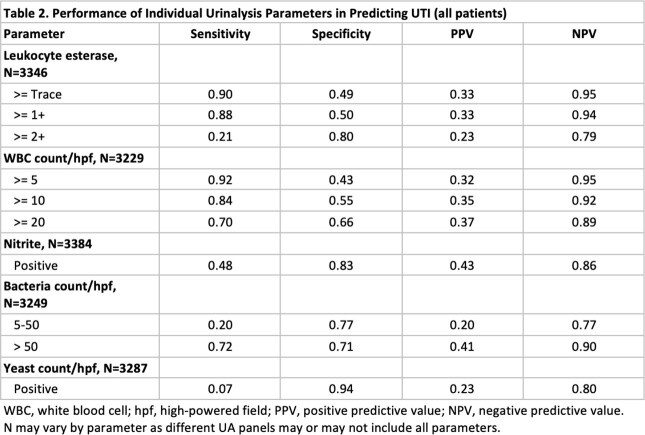

**Figure:**
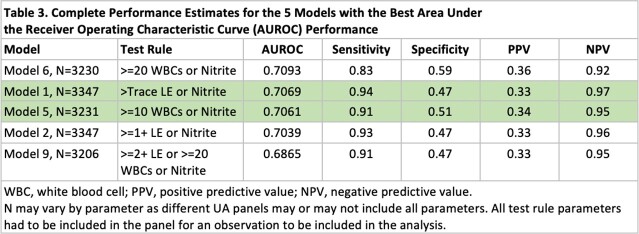

**Conclusion:**

Our review of laboratory and symptom data from a diverse population dataset revealed that combined UA parameters were better at predicting UTI, but performance of UA parameters differs based on age, sex, and urine culture thresholds. Our approach highlights the need to move away from a one-size fits all approach to using population specific UA cut-offs for patients with UTI symptoms.

**Figure:**
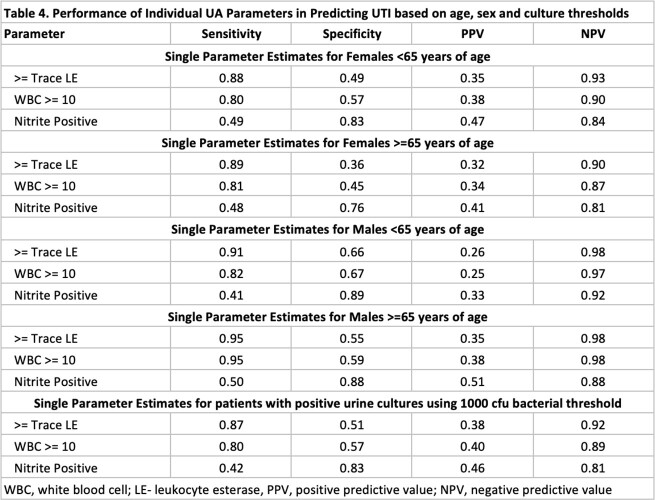

**Disclosures:**

**Sonali Advani, MBBS, MPH, FIDSA**, bioMérieux: Advisor/Consultant|GlaxoSmithKline: Advisor/Consultant **Nicholas A. Turner, MD, MHSc**, PDI: Research contract to assess efficacy of cleaning agent

